# Design of a Sensor Based on Plastic Optical Fibre (POF) to Measure Fluid Flow and Turbidity

**DOI:** 10.3390/s90503790

**Published:** 2009-05-19

**Authors:** Pedro Aiestaran, Jon Arrue, Joseba Zubia

**Affiliations:** 1 University of the Basque Country, Department of Electronics and Telecommunications, San Sebastián, Spain; 2 University of the Basque Country, Department of Electronics and Telecommunications, Bilbao, Spain; E-Mails: jon.arrue@ehu.es; joseba.zubia@ehu.es

**Keywords:** plastic optical fibre, flow and turbidity measurement sensor

## Abstract

Although many optical fibre applications are based on their capacity to transmit optical signals with low losses, it can also be desirable for the optical fibre to be strongly affected by a certain physical parameter in the environment. In this way, it can be used as a sensor for this parameter. There are many strong arguments for the use of POFs as sensors. In addition to being easy to handle and low cost, they demonstrate advantages common to all multimode optical fibres. These specifically include flexibility, small size, good electromagnetic compatibility behaviour, and in general, the possibility of measuring any phenomenon without physically interacting with it. In this paper, a sensor based on POF is designed and analysed with the aim of measuring the volume and turbidity of a low viscosity fluid, in this case water, as it passes through a pipe. A comparative study with a commercial sensor is provided to validate the proven flow measurement. Likewise, turbidity is measured using different colour dyes. Finally, this paper will present the most significant results and conclusions from all the tests which are carried out.

## 1. Introduction

Historically, the idea of optical communications has been around for more than a century, but it has only been possible to put the idea into practice in the last few decades. In the first known optical application, Alexander Graham Bell obtained a patent for his elemental photophone in 1880. However, like many other ideas, this was ahead of its time and the technology to develop it was not in place.

In the field of communications, there were two important developments that represented great advances in the field. The first occurred in 1960 with the invention of the laser. The second development involved obtaining a very pure glass fibre with very low attenuation.

Over the past 30 years a new type of optical fibre has been researched, namely the plastic optical fibre (POF). Since Koike developed graded-index plastic optical fibres in 1990, later obtaining low attenuation perfluorinated fibres (in 1996) [1], plastic optical fibres have received a lot of interest, and a great number of applications is expected to be developed over the next few years.

Some of the most important advantages of an optical fibre are: low loss, high bandwidth, small size, low cost, great flexibility, high resistance to radiation, high stability over a wide temperature range, easy handling and electrical isolation.

In the field of sensors, numerous types of products based on POFs have been commercialized [2,3], for example scanning heads, shape-defect detectors used in bottling plants, and liquid level detectors. In addition, by using conventional POFs, it is possible to measure parameters such as nuclear radiation, humidity, temperature, concentrations, displacements, toxicity and so on [4-6].

Let us first review the basic concepts involved in measuring flow rate and turbidity. Flow rate is defined as the volume of fluid per unit time that passes through a pipe, and it can be calculated as the product of the fluid speed and the cross-section of the pipe. The most commonly used units to express the flow rate are litre/minute and m^3^/hour. The flow rate measurements can be taken using a variety of traditional technologies:

- By differential pressure
- By mechanical driver
- By electromagnetic driver
- By ultrasonic waves

Regarding turbidity, the most commonly used unit is the NTU, or nephelometric turbidity unit, which is calibrated on the basis of health considerations [7]. Water for human consumption should have turbidity below 5 NTU for aesthetic reasons. Several methods may be used to measure water turbidity, but only two of them, nephelometry and turbidimetry, form the basis of present standard methods [8]. Historically, turbidity has been measured in drinking water using the Jackson candle turbidimeter. This is a visual method in which the sample is poured into a calibrated tube and the turbidity is read when the flame of a candle under the bottom of the tube disappears from view. A depth of 21.5 cm corresponds to 100 Jackson turbidity units (JTU) [9]. As an alternative, turbidimeters can be calibrated in terms of the concentration (in mg/L) of suspended solids causing turbidity (a gravimetric definition). A Jackson candle turbidimeter can only be applied to turbidities greater than 25 JTU, so it is not suitable for monitoring drinking water. Improved instruments that use electrical light sources and mirror optics, such as the Patterson turbidimeter, can measure lower values. The current method of choice for measuring turbidity is the nephelometric method [10]. These turbidimeters measure the intensity of light scattered at 90° as the beam of light passes through a water sample. It will be necessary to calibrate the measured values because of differences in the physical design of turbidimeters. Parameters such as the light source, sample geometry or detector geometry are therefore defined for calibration purposes. This paper presents a sensor to measure flow rate and turbidity. This sensor is based on using plastic optical fibre and making the most of its excellent properties.

### 2. Operating Principle

The operating principle is based on a rotating propeller placed in a pipe, perpendicular to liquid movement. When the liquid crosses the blades it creates rotation power that turns the rotor and its speed is directly proportional to the flow. An optical transducer using plastic optical fibre generates a pulse train, as a function of the rotor speed. The number of pulses per time unit is a function of the flow rate. On the other hand, the ray of light that crosses the pipe is sensitive to its transmission medium. This means that the higher the degree of fluid turbidity, the lower the amplitude of the received signal, meaning that the height of the pulses is a function of the fluid's turbidity.

A general, commercially sold, rotating propeller was used as the mechanical part to produce this sensor. Using plastic optical fibre to send and receive the optical signals generated by the rotating propeller as it turns helps us to classify this type of sensor as a sensor based on plastic optical fibre.

Measurement accuracy is based on achieving a steady and undisturbed flow with a fully developed turbulent flow profile. There are two basic types of flow profile: turbulent and laminar. Turbulent flow exists when the speed of the fluid in the pipe is practically constant across its entire width. This is typical of low viscosity fluids; like water, flowing at high velocity. Laminar flow exists when the speed of the fluid in the centre of the pipe is greater than its speed at the outer edge, near the pipe wall. This is typical of high viscosity fluids flowing at low velocity. Because the meter commonly only measures the flow near the pipe wall (particularly in larger pipe sizes), a constant velocity across the flow stream is required [11-12].

To determine the type of flow in an installation, the dimensionless REYNOLDS NUMBER, RN, is used:

(1) $$RN=\frac{D.V.\rho }{\mu }$$

where *D* is the pipe diameter in inches; *V* the fluid velocity; *ρ* the fluid density and *μ* the fluid viscosity.

Flow conditions with a Reynolds Number greater than 4,000 represent a fully developed turbulent flow. A Reynolds Number under 2,000 is laminar flow. The meter requires a Reynolds number greater than 4,000 to maintain accuracy.

**Figure 1.**
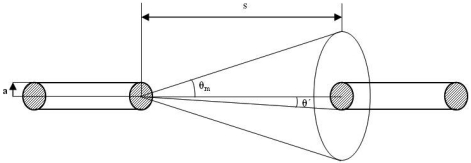
LED emits light through the s-gap.

The coupling between two fibres is an important factor to take into consideration. Considering *Io* as the optical power *dP* contained in a solid angle *dΩ* radiated by an elementary source area [13-14], the solid angle *dΩ* defines a cone centred in the direction *(θ,θ′,Φ)* where *θ,θ′* and *Φ* are angles relative to the normal of the elementary source area located at centre of the fibre, as shown in Figure 1.

The total power radiated by the source fibre is:

(2) $$P_{\text{out}}=\int_{0}^{2\pi }\int_{0}^{\theta_{m}}\left(Io.\cos\theta \right).\sin\theta .d\theta .d\varphi_{s}=\pi .Io.\sin^{2}\theta_{m}$$

and the power arriving in the receiver fibre is:

(3) $$P_{in}=\int_{0}^{2\pi }\int_{0}^{\theta^{\prime }}\left(Io.\cos\theta \right).\sin\theta .d\theta .d\varphi_{s}=\pi .Io.\sin^{2}\theta^{\prime }$$

where *s* is the separation between fibres and *a* is the radius of the fibre. The ratio between both powers is:

(4) $$\frac{P_{in}}{P_{\text{out}}}=\frac{\sin^{2}\theta^{\prime }}{\sin^{2}\theta_{m}}=\frac{a^{2}}{{\left(a+s.\tan\theta \right)}^{2}}.\frac{s^{2}+{\left(a+s\tan\theta \right)}^{2}}{s^{2}+a^{2}}$$

The loss R due to Fresnel's reflections must also not be forgotten. This phenomenon is due to the reflection when an optical radiation passes from one medium to another with a different refractive index. Fresnel's reflections are given by:

(5) $$R_{12}={\left[\frac{n_{1}-n_{2}}{n_{1}+n_{2}}\right]}^{2}$$

(6) $$R_{12}\cdot R_{21}=R^{2}$$

where n_1_ is the fibre (core) refractive index and n_2_ is the liquid (water) refractive index. In this case, loss depends on *(1-R)^2^* because Fresnel's reflections happen twice [15].

(7) $${\left[\frac{\text{Pout}}{\text{Pin}}\right]}_{F}={\left(1-R\right)}^{2}$$

In our tests, the parameters used are *s* = 15 mm; *α_a_* = 30°; *a* = 0,365mm; *n_1_* = 1.49; *n_2_* = 1.33.

## 3. System description

Basically, the designed system comprises two different parts:

### 3.1. Mechanical part

**Figure 2.**
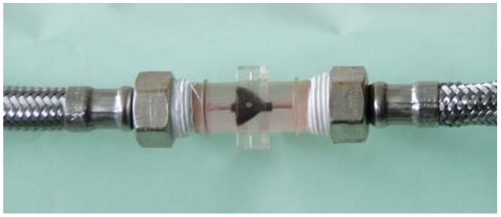
Rotating propeller.

The mechanical part is composed of a two-blade rotating propeller turning around its axis as the fluid passes through the pipe, as shown in Figure 2. This rotation affects light coupling from one POF to another when both are placed in line a short distance apart, with the propeller in between. This set-up generates an optical pulse train when the relative position of the propeller changes in relation to the emitted light beam. This pulse train is then sent to an optoelectronic system by means of the second POF.

### 3.2. Optoelectronic Part

This converts the optical signal into electrical information. The frequency of the generated pulse train is intrinsically related to the speed of the fluid through the pipe. This speed multiplied by the pipe section will give us the volume of fluid per unit time, or flow rate. On the other hand, the amplitude of the pulse train is a function of the turbidity of the fluid.

Since the system measures both flow rate and turbidity, it was necessary to design two independent circuits. The first measures the flow rate, testing the frequency of the generated pulse train. This circuit is composed of a Contrinex LFK-3030 photoelectric proximity switch, and a voltage amplifier. The photoelectric proximity switch device includes a Schmitt Trigger circuit providing a regular pulse train which is suitable to obtain the flow rate measurement, although the pulse amplitude is constant and not valid to measure turbidity. The second circuit measures turbidity, checking the amplitude of the signal. Measuring this amplitude required a Plastic Fibre Optic IF-E96 red LED whose output spectrum is produced by a GaAlAs die which peaks at 660 nm, a Plastic Fibre Optic Photodiode IF-D91 whose optical response stretches from 400 to 1,100 nm, and a transimpedance amplifier followed by a peak detector circuit. A subsequent amplifier is used to condition this signal by isolating the circuit output. Moreover, a variable voltage divider is set up to adjust output values according to the display values required. In addition, an electronic circuit has been prepared to display the two totally independent magnitudes in a Powertip LCD PC1602-Q.

**Figure 3.**
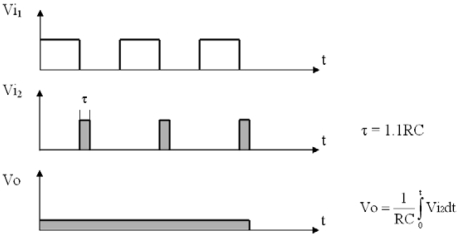
Trigger activated by down edges.

The sensor propeller is installed in the pipe where the fluid is circulating. When the two blades rotate, they interrupt the light path, and intermittent pulses are generated. The rotation speed depends on the flow rate. A trigger control circuit has been designed to measure the width of all pulses as accurately as possible, *V_i1_*. It is triggered by down edges and it employs the NE555 IC set in the measuring circuit input, *V_i2_*. This allows us to obtain pulses with a constant width, which will be connected to a measuring circuit, as shown in Figure 3.

After obtaining this constant width pulse train, a simple RC circuit is integrated and a continuous voltage value is achieved, which shows the frequency behaviour of the signal in real time. This voltage is given by:

(8) $$V_{0}=\frac{1}{R\cdot C}\int_{0}^{T}Vi2dt$$

Using the theory of dimensionless parameters, it can be proved that a linear relationship exists between the flow rate through the pipe and the angular speed of the propeller, as follows:

(9) $$J_{R}=\frac{Q}{\omega \cdot D^{3}}=K_{1}\Rightarrow Q=K_{1}\cdot D^{3}\cdot \omega$$

(10) $$Q=K_{2}\cdot \omega =0,0149\cdot \omega$$

where J_R_ is a flow parameter, Q is the flow rate, D is the pipe diameter, ω is the angular speed and K_1_, K_2_ are constants.

**Figure 4.**
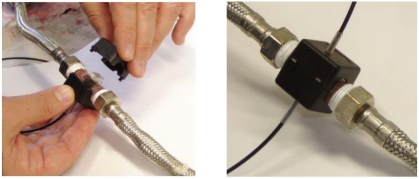
Mechanical model assembly.

Finally, the signal is conditioned by setting up an adjustable gain voltage amplifier circuit using a potentiometer, with the aim of calibrating the *Vo* signal to the correspondent *Q* value, after having been isolated from the rest of circuit. The mechanical assembly can be seen in Figure 4.

**Figure 5.**
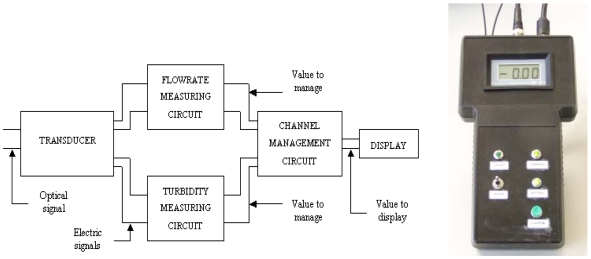
Complete block diagram and unit control for the sensor.

A channel management circuit has been designed to avoid using two displays to visualise two different kinds of information. Using a switch, one kind of information or another can be displayed. An analogue multiplexer has been used for this design. The measurement circuit outputs for turbidity and flow rate are connected to the management circuit. A switch is connected to one RS flip-flop and, after that, its output is connected to a J-K latch, and this generates a control signal. By means of this generated control signal, the displayed signal is connected to the LCD display circuit, as shown below. The complete block diagram and the unit control for the sensor appear in Figure 5.

## 4. Results

The results obtained will be classified into two groups: flow measuring tests and turbidity measuring tests.

### 4.1. Flow Rate Measuring Tests

**Figure 6.**
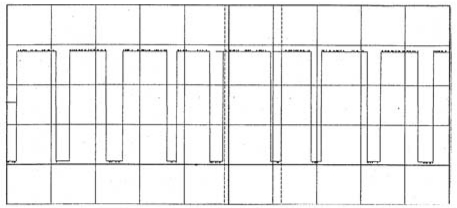

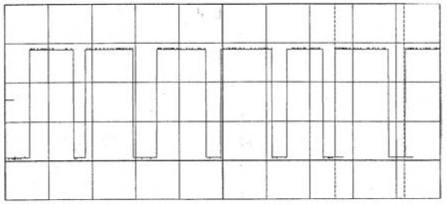

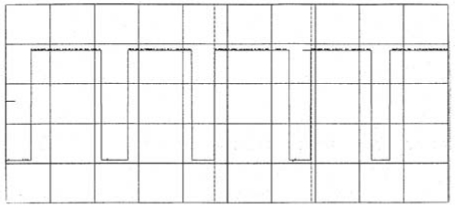

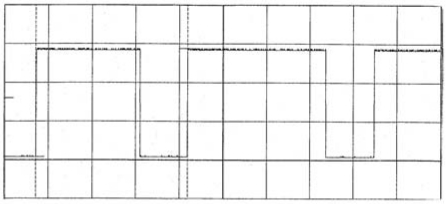

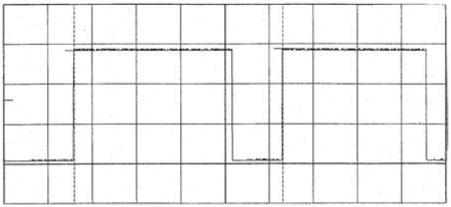
Figure 6-a. Frequency 39,063 Hz. Figure 6-b. Frequency 31,250 Hz. Figure 6-c. Frequency 22,727 Hz. Figure 6-d. Frequency 14,388 Hz. Figure 6-e. Frequency 10,604 Hz.

Several tests have been run with the aim of obtaining a comparative study for the designed system and the GPI commercial flowmeter. Different shape signals can be seen in Figures 6-a, 6-b, 6-c, 6-d and 6-e, corresponding to specific tests. The generated pulse train and the frequency value appear in each graph.

The results of the comparative study between a commercial flowmeter and the POF-based system designed are shown in Table 1. The measurements have been obtained using an analog-digital HAMEG HM1507 oscilloscope and the SP107 V1.61 software. These measurements were taken every minute and for a total time of 5 minutes. Furthermore, the tests have been carried out with five different flow rates and, the flow rate columns show the different measurements obtained with the commercial flowmeter and the designed system. The corresponding values obtained with the POF flowmeter are expressed in value rates due to the fact that the designed hardware sensitivity gives us flow rate variations to the second decimal.

### 4.2. Turbidity Measurement Tests

Red (660 nm), green (530 nm) and infrared (880 nm) LEDs have been used to measure turbidity. The optical fibre used is a PMMA POF with a core diameter of 730 μm; the optical fibre lengths for emission and reception are 1.5 m. At the request of a company working with different kinds of dyes, the turbidity tests were carried out using a volume of 10 litres of water, with different dyes, which affect the signal amplitude value. Red, black and blue commercial dyes were used.

The signals obtained at the corresponding photosensors are proportional to the light power received, and then processed appropriately. A first transimpedance stage is followed by a voltage amplification stage. The results obtained are presented in the following Figures.

**Figure 7.**
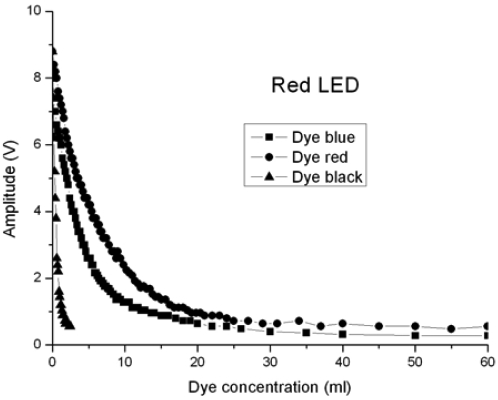
Tests with a red LED where λ = 660 nm.

Figure 7 shows a comparative study of three graphs obtained with the red, black and blue dyes, using a red LED where λ = 660 nm. The vertical axis shows the signal amplitude expressed in volts, whereas the horizontal axis shows the concentration dye expressed in mL per 10 litres of water. The greatest loss takes place with the black dye, followed by the blue dye and finally the red one.

**Figure 8.**
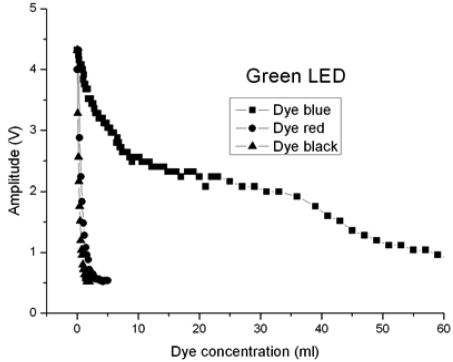
Tests with a green LED where λ = 530 nm.

Figure 8 shows the comparison between the red, black and blue dyes, using a green LED where λ = 530 nm. The greatest loss takes place with the black dye, followed by the red dye and finally the blue one.

**Figure 9.**
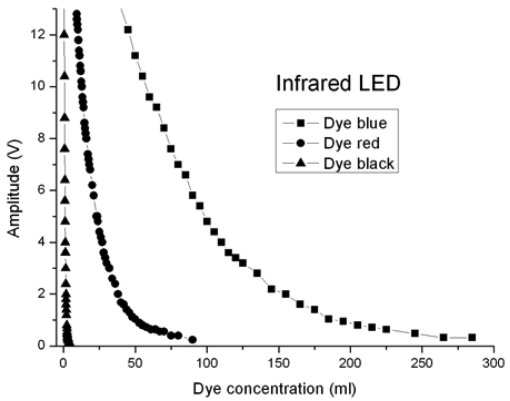
Tests with a infrared LED where λ = 880 nm.

Figure 9 shows the comparison between the red, black and blue dyes, using an infrared LED where λ = 880 nm. The greatest loss takes place with the black dye, followed by the red dye and finally the blue one.

## 5. Conclusions

A sensor that allows us to measure flow and turbidity without physical interaction with the liquid being measured has been designed, constructed and tested. It is low cost and fast and simple to implement, which makes it very useful for domestic and industrial applications.

The designed system takes flow rate measurements which are correct as required, meaning, correct flow rate measurements after making the suitable comparisons with a commercial flowmeter.

Turbidity is a parameter whose significance is, to a large extent, dependent on measurement technique, detector geometry, and on factors such as the size, shape, refractive index of the dye or particles and the wavelength of the incident light. It has therefore been necessary to design a specific system taking the application type into account.

Blue, red and black dyes were used to simulate turbidity. The light sources were red, green and infrared LEDs. For the red LED, the best result was obtained with the red dye. The green LED case gives the best solution with the blue dye, and the infrared LED gives the best result with the blue dye. Bearing in mind all the cases tested, the best combination to make a turbidity sensor is the blue dye with the infrared LED.

## Figures and Tables

**Table 1.** Summary table giving different flow rate measurements.

	Stopcock open (f = 40Hz)		Stopcock semiopen I (f = 32Hz)		Stopcock semiopen II (f = 23Hz)		Stopcock semiclosed I (f = 14Hz)		Stopcock semiclosed II (f = 11Hz)	
N° min.	GPI (l/m)	F.O.P. (l/m)	GPI (l/m)	F.O.P. (l/m)	GPI (l/m)	F.O.P. (l/m)	GPI (l/m)	F.O.P. (l/m)	GPI (l/m)	F.O.P. (l/m)
1	3.75	3.70-3.80	3.00	2.95-3.05	2.17	2.10-2.20	1.21	1.20-1.28	0.84	0.90- 0.93
2	7.47 (3.72)	3.70-3.80	6.02 (3.02)	2.95-3.05	4.34 (2.17)	2.10-2.20	2.43 (1.22)	1.20-1.28	1.68 (0.84)	0.90- 0.93
3	11.22 (3.75)	3.70-3.80	9.04 (3.02)	2.95-3.05	6.47 (2.13)	2.10-2.20	3.64 (1.21)	1.20-1.28	2.53 (0.85)	0.90- 0.93
4	14.94 (3.72)	3.70-3.80	12.00 (2.96)	2.95-3.05	8.60 (2.13)	2.10-2.20	4.85 (1.21)	1.20-1.28	3.37 (0.84)	0.90- 0.93
5	18.65 (3.71)	3.70-3.80	15.00 (3.00)	2.95-3.05	10.75 (2.15)	2.10-2.20	6.09 (1.24)	1.20-1.28	4.21 (0.84)	0.90- 0.93
